# Prostaglandin E _2_ and the protein kinase A pathway mediate arachidonic acid induction of *c-fos* in human prostate cancer cells

**DOI:** 10.1054/bjoc.2000.1143

**Published:** 2000-05-18

**Authors:** Y Chen, M Hughes-Fulford

**Affiliations:** Laboratory of Cell Growth, University of California, San Francisco and Veterans Affairs Medical Center, Mail Code 151F, 4150 Clement Street, San Francisco, CA, 94121, USA

**Keywords:** prostate cancer *c-fos*, arachidonic acid, prostaglandin E _2_, protein kinase, gene expression, prostaglandin receptor, EP4

## Abstract

Arachidonic acid (AA) is the precursor for prostaglandin E _2_(PGE _2_) synthesis and increases growth of prostate cancer cells. To further elucidate the mechanisms involved in AA-induced prostate cell growth, induction of *c-fos* expression by AA was investigated in a human prostate cancer cell line, PC-3. *c-fos* mRNA was induced shortly after addition of AA, along with a remarkable increase in PGE _2_ production. *c-fos* expression and PGE _2_ production induced by AA was blocked by a cyclo-oxygenase inhibitor, flurbiprofen, suggesting that PGE _2_ mediated *c-fos* induction. Protein kinase A (PKA) inhibitor H-89 abolished induction of *c-fos* expression by AA, and partially inhibited PGE _2_ production. Protein kinase C (PKC) inhibitor GF109203X had no significant effect on *c-fos* expression or PGE _2_ production. Expression of prostaglandin (EP) receptors, which mediate signal transduction from PGE _2_ to the cells, was examined by reverse transcription polymerase chain reaction in several human prostate cell lines. EP4 and EP2, which are coupled to the PKA signalling pathway, were expressed in all cells tested. Expression of EP1, which activates the PKC pathway, was not detected. The current study showed that induction of the immediate early gene *c-fos* by AA is mediated by PGE _2_, which activates the PKA pathway via the EP2/4 receptor in the PC-3 cells. © 2000 Cancer Research Campaign


*British Journal of Cancer* (2000) **82**(12), 2000–2006
© 2000 Cancer Research Campaign
doi: 10.1054/ bjoc.2000.1143, available online at http://www.idealibrary.com on IDEAL®

### Prostaglandin $E_2$ and the protein kinase A pathway mediate arachidonic acid induction of *c-fos* in human prostate cancer cells

**Y Chen and M Hughes-Fulford**

Laboratory of Cell Growth, University of California, San Francisco and Veterans Affairs Medical Center, Mail Code 151F, 4150 Clement Street, San Francisco, CA 94121, USA

**Summary** Arachidonic acid (AA) is the precursor for prostaglandin $E_2$ ($PGE_2$) synthesis and increases growth of prostate cancer cells. To further elucidate the mechanisms involved in AA-induced prostate cell growth, induction of *c-fos* expression by AA was investigated in a human prostate cancer cell line, PC-3. *c-fos* mRNA was induced shortly after addition of AA, along with a remarkable increase in $PGE_2$ production. *c-fos* expression and $PGE_2$ production induced by AA was blocked by a cyclo-oxygenase inhibitor, flurbiprofen, suggesting that $PGE_2$ mediated *c-fos* induction. Protein kinase A (PKA) inhibitor H-89 abolished induction of *c-fos* expression by AA, and partially inhibited $PGE_2$ production. Protein kinase C (PKC) inhibitor GF109203X had no significant effect on *c-fos* expression or $PGE_2$ production. Expression of prostaglandin (EP) receptors, which mediate signal transduction from $PGE_2$ to the cells, was examined by reverse transcription polymerase chain reaction in several human prostate cell lines. EP4 and EP2, which are coupled to the PKA signalling pathway, were expressed in all cells tested. Expression of EP1, which activates the PKC pathway, was not detected. The current study showed that induction of the immediate early gene *c-fos* by AA is mediated by $PGE_2$, which activates the PKA pathway via the EP2/4 receptor in the PC-3 cells. © 2000 Cancer Research Campaign

**Keywords**: prostate cancer; *c-fos*; arachidonic acid; prostaglandin $E_2$; protein kinase; gene expression; prostaglandin receptor; EP4

Dietary fatty acid intake is associated with the risk of development and progression of prostate, colon and breast cancer (Marnett, 1992; Rose, 1997). In vitro and in vivo studies suggest that the availability of polyunsaturated fatty acids contributes to increased cancer cell growth, and that inhibitors of the eicosanoids synthesis pathway inhibit cell proliferation (Rose and Connolly, 1991; Connolly et al, 1997).

Arachidonic acid (AA) is derived from the essential polyunsaturated fatty acid, linoleic acid, which is commonly available in dietary fat. It is the major precursor of biologically active eicosanoids, which include prostaglandins, thromboxanes and leukotrienes. Among these is prostaglandin $E_2$ ($PGE_2$), a product catalysed by the key enzyme cyclo-oxygenase (COX, EC 1.14.99.1). Two isoforms of COX exist in human prostate cells. COX-1 is a constitutively expressed exzyme, whereas COX-2 is inducible. O'Neill and Ford-Hutchinson (1993) have reported that the human prostate cells express similar amounts of COX-1 and COX-2. In human prostate tissues, $PGE_2$ is the only significant eicosanoid produced (Chaudry et al, 1994). $PGE_2$ induces a variety of cell responses depending on the tissue type and the receptors involved, such as immune regulation (Monick et al, 1987; Juzan et al, 1992; Marnett, 1992), smooth muscle contraction (Coleman and Kennedy, 1985) and regulation of water re-adsorption in kidney (Smith, 1989). Previous studies with osteoblasts and prostate cancer cells have demonstrated that $PGE_2$ stimulates cell growth (Raisz et al, 1993; Tjandrawinata et al, 1997).

Studies from this laboratory indicate that AA stimulates growth of a prostate cell line, PC-3 (manuscript submitted). Because of the role of $PGE_2$ in prostate cancer cell growth, we hypothesized that AA had the stimulatory effect on the prostate cancer cells through the activity of its metabolite $PGE_2$, and aimed to understand the signal transduction events following AA administration which lead to the growth of the cells. After being synthesized and exported out of the cells, $PGE_2$ exerts its functions by interacting with the $PGE_2$ receptors (EPs), which are 7-domain transmembrane cell surface receptors. Four subtypes of EP receptors have been identified and characterized. These receptors are coupled to G proteins, and activate or inhibit second messenger systems inside the cell. EP1 causes influx of $Ca^{2+}$ and activation of protein kinase C (PKC); receptors EP2 and EP4 activate the adenylate cyclase which increases cellular cyclic AMP level and activates protein kinase A (PKA); EP3 signals primarily through an inhibitory G protein to decrease intracellular cyclic AMP levels (Negishi et al, 1995).

Despite the important association of AA and $PGE_2$ with prostate cancer, the signals mediating the biological functions of these molecules in prostate cancer cells are not fully understood. The signalling pathways mediating AA- or $PGE_2$-induced cell growth or expression of growth-related proto-oncogenes have been investigated in a number of studies with varying conclusions. In bone cells, a PKA-mediated mechanism has been suggested (Fitzgerald et al, 1999; Weinreb et al, 1999). A study of smooth muscle cells demonstrated a role for PKC as a mediator of AA-induced *c-fos*

*Received 29 November 1999*
*Revised 2 February 2000*
*Accepted 5 February 2000*

*Correspondence to:* M Hughes-Fulford expression (Rao et al, 1993). In the Swiss 3T3 fibroblast cells, however, there are conflicting data on whether PKC or PKA is involved (Kacich et al, 1988; Mehmet et al, 1990; Danesch et al, 1994). Our results indicate that the PKA-dependent pathway via the EP4 receptor is responsible for mediating AA-induced *c-fos* expression in human prostate cancer cells.

### MATERIALS AND METHODS

### Materials

AA, flurbiprofen, and 12-O-tetradecanoylphorbol 13-acetate (TPA) were purchased from Sigma (St Louis, MO, USA). PKC inhibitor GF109203X and PKA inhibitor H-89 were from BIOMOL (Plymouth Meeting, PA, USA). PGE$_2$ was from Oxford (Oxford, MI, USA). RPMI-1640 medium, L-glutamine, and antibiotics were from UCSF Cell Facility (San Francisco, CA, USA). Fetal calf serum (FCS) was purchased from Hyclone Laboratories (Logan, UT, USA).

### Cell culture

The PC-3, DU145 and LNCaP human prostate cancer cell lines were cultured in complete RPMI-1640 medium supplemented with 10% FCS, 2 mM L-glutamine, 25 mM glucose, 1 mM pyruvic acid, 100 units ml$^{-1}$ penicillin, 100 μg ml$^{-1}$ streptomycin and 0.25 μg ml$^{-1}$ amphotericin B. The PrEC normal human prostate epithelial cells (Clonetics, San Diego, CA, USA) were maintained in the PrEGM medium (Clonetics). All cells were cultured with 5% carbon dioxide at 37°C. For *c-fos* expression experiments, cells were grown in complete RPMI medium with 5% FCS for 2 days, and then 1% FCS for 24 h before cells were harvested for RNA isolation.

### PCR primers

Specific polymerase chain reaction (PCR) primers (see Table 1) were designed by MHF. The primer pairs span introns of the genes, so that the reverse transcription PCR (RT-PCR) products may be distinguished from any possible PCR products resulted from contaminating genomic DNA. Primers were synthesized by Operon Technologies (Alameda, CA, USA).

### Semi-quantitative RT-PCR

Semi-quantitative kinetic RT-PCR was used for analysis since this is the most versatile and inexpensive method available. The PCR efficiency of the reactions was checked by examining the slopes of the PCR product curves and internal standards of housekeeping genes from the same samples. RNA was isolated from cells grown in 6-well plates using the TriReagent (Sigma) as recommended by the company. RNA was quantified in the GeneQuant spectrophotometer (Pharmacia, Piscataway, NJ, USA). The RNA PCR kit from PE Applied Biosystems (Foster City, CA, USA) was used for reverse transcription and PCR following procedures recommended by the manufacturer. An aliquot of 1.5 μg RNA was reverse transcribed in 30 μl of buffer. Five microlitres of the reverse transcription reaction was PCR-amplified using specific primers. A temperature cycle in PCR was 94°C for 1 min 40 s, 63°C for 1 min 10 s and 72°C for 1 min 40 s. PCR was carried out in a Robocycler 40 (Stratagene, San Diego, CA, USA) for various cycle numbers depending on the primers used (the cycle numbers are described in the legends for Tables and Figures). PCR cycle numbers were maintained within a linear amplification range. The PCR products were separated by electrophoresis in 2% agarose gels. The gels were photographed and DNA bands of interest were scanned at 400 dpi with an Epson Perfection 636 scanner. Areas and densities of the DNA bands were determined using the SigmaGel software (Sigma).

### PGE$_2$ assay

An aliquot of culture medium was collected and frozen at –70°C before the cells were harvested for RNA isolation. PGE$_2$ levels in the culture medium samples were determined using a PGE$_2$ monoclonal enzyme immunoassay kit (Cayman Chemical, Ann Arbor, MI, USA), following the protocols recommended by the company. A Dynatech MR5000 microplate reader (Dynatech Laboratories, Chantilly, VA, USA) was used to read the assay results. Data were analysed using the BioLinx 2.0 software (Dynatech).

### Statistical analysis

For each treatment in the experiments, three independent samples were seeded, treated and their RNA or media samples analysed separately. Mean and standard deviation (s.d.) of the three samples

**Table 1** Primers used in RT-PCR

| Gene | Orientation | Sequence | Product size (bp) |
|---|---|---|---|
| *c-fos* | Sense | 5′ GAATAAGATGGCTGCAGCCAAATGCCGCAA | 236 |
| | Anti-sense | 5′ CAGTCAGATCAAGGGAAGCCACAGACATCT | |
| Cyclophilin | Sense | 5′ CGTCTCCTTTGAGCTGTTTGCAGAC | 628 |
| | Anti-Sense | 5′ CATAATCATAAACTTAACTCTGCAATCCAGC | |
| *EP1* | Sense | 5′ CCACCACCTTCCTGCTGTTCG | 1037 |
| | Anti-sense | 5′ GGTGGGCTGGCTTAGTCGTTG | |
| *EP2* | Sense | 5′ CCTGTTGGGCACTTTGTTGGT | 784 |
| | Anti-sense | 5′ GGGGTCTAGGATGGGGTTCAC | |
| *EP3* | Sense | 5′ CGCCTCAACCACTCCTACACA | 837 |
| | Anti-sense | 5′ GCAGACCGACAGCACGCACAT | |
| *EP4* | Sense | 5′ AGGATTGCTTCTGTGAACCCCAT | 344 |
| | Anti-sense | 5′ GAGGTGGTGTCTGCTTGGGTCAG | |

© 2000 Cancer Research Campaign are shown in the figures. One-way analysis of variance (ANOVA) was performed using the SigmaStat (Sigma) software to obtain the *P*-values for comparison between treatments.

### RESULTS

### AA transiently increases *c-fos* mRNA expression in PC-3 cells

A previous study from this laboratory demonstrated that AA induces *c-fos* expression in the PC-3 human prostate cancer cells (manuscript submitted). In this study, AA was added to the PC-3 cells at a concentration of 1 μg $ml^{-1}$ since previous work demonstrated that *c-fos* expression can be up-regulated with as low as 0.1 μg $ml^{-1}$ of AA, and that the effect is near maximum when the AA concentration is at 0.5 μg $ml^{-1}$. Fifteen, 30, 60 and 180 min after addition of AA, total RNA was isolated from the cells and was subjected to RT-PCR analysis to observe level of *c-fos* mRNA. Table 2 summarizes the relative level of *c-fos* product after being normalized to the internal control, cyclophilin. *c-fos* message was induced as early as 15 min after AA addition to cell culture, and was greater than 1.9-fold higher than the non-treated control at 1 h. Three hours after AA addition, *c-fos* mRNA level began to decrease, suggesting that *c-fos* induction was transient and was regulated by other downstream events. Based on these observations, cells and medium samples were collected 1 h after AA was added to culture media for all subsequent AA-induction experiments.

### AA-induced *c-fos* expression is mediated by $PGE_2$

Because AA is the precursor of $PGE_2$, it was speculated that the AA-induced *c-fos* expression was due to increased $PGE_2$ production by the PC-3 cells. Culture medium samples collected from the experiment presented in Table 2 were analysed for their $PGE_2$ levels. As expected, $PGE_2$ production of the PC-3 cells increased shortly after its precursor, AA, became available to the cells and $PGE_2$ level remained elevated for at least 3 h (Table 2).

In order to confirm that $PGE_2$ mediated AA-induced *c-fos* expression, we blocked $PGE_2$ synthesis with a non-steroidal anti-inflammatory drug (NSAID). Flurbiprofen, which suppresses cyclo-oxygenases and subsequently $PGE_2$ production, was added to the cells before the AA treatment. As shown in Figure 1A, *c-fos* message induction by AA was inhibited by flurbiprofen, suggesting an important role for $PGE_2$ production in this event. Increased *c-fos* expression in PC-3 cells was similarly achieved by $PGE_2$ treatment (Figure 1B). The effect of flurbiprofen on $PGE_2$ production by PC-3 cells was measured. $PGE_2$ level of the culture media was increased more than sevenfold by AA, but this increase was abolished by flurbiprofen treatment (Figure 2). These data strongly suggest that AA increases *c-fos* expression via a $PGE_2$ mechanism.

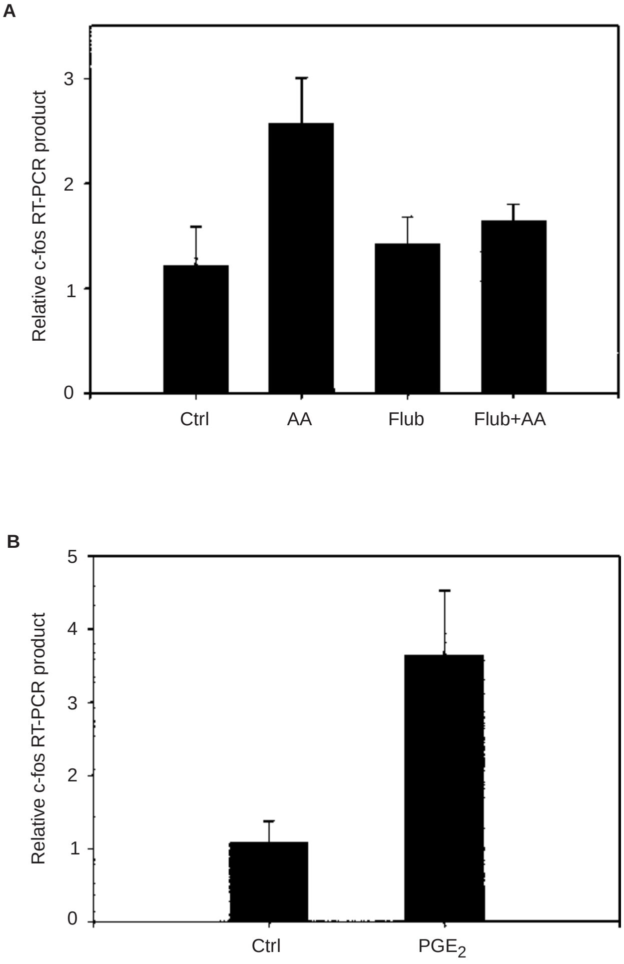

**Figure 1** Effect of NSAID and $PGE_2$ on AA-induced *c-fos* expression in PC-3 cells. Cells were treated as indicated before harvested for total RNA isolation and subsequent RT-PCR. Amount of *c-fos* RT-PCR product was normalized to that of cyclophilin product. Mean ± s.d. of three independent samples is shown in this bar graph. Ctrl = control; Flub = flurbiprofen. (**A**) Flurbiprofen (30 μM) was added to the cells 3 h before harvest and AA (1 μ $ml^{-1}$) was added 1 h before harvest. *c-fos* induction by AA was statistically significant ($P = 0.004$) in absence of flurbiprofen, but was not significant ($P = 0.840$) in presence of flurbiprofen. (**B**) $PGE_2$ (4 μg $ml^{-1}$) was added to the cells 30 min before harvest. Induction by $PGE_2$ was significant ($P = 0.009$)

**Table 2** AA-induced *c-fos* mRNA expression and $PGE_2$ production in PC-3 cells

| Incubation time with AA | Relative *c-fos* product | $PGE_2$ concentration (pg $ml^{-1}$) |
|---|---|---|
| Control | 2.81 ± 0.26 | 80 ± 34 |
| 15 min | 3.59 ± 1.09 | 361 ± 107 |
| 30 min | 4.42 ± 0.47 | 402 ± 18 |
| 1 h | 5.46 ± 0.53 | 425 ± 52 |
| 3 h | 3.62 ± 0.69 | 558 ± 131 |

Total RNA was isolated from the PC-3 cells after incubation with AA (1 μg $ml^{-1}$) for a time period indicated in the Table. RT-PCR with *c-fos* and cyclophilin primers was performed on the RNA samples. PCR reactions with *c-fos* and cyclophilin primers had 29 and 19 temperature cycles respectively. The amount of *c-fos* RT-PCR product was normalized to that of the cyclophilin product. Culture medium samples from the same experiment was assayed for $PGE_2$ level. The numbers represent mean ± s.d. of three independent samples.

### AA-induced *c-fos* expression is dependent on the PKA signalling pathway

The EP receptors for $PGE_2$ are coupled to either PKC or PKA pathways. To determine which EP receptor signalling pathway is

 © 2000 Cancer Research Campaign

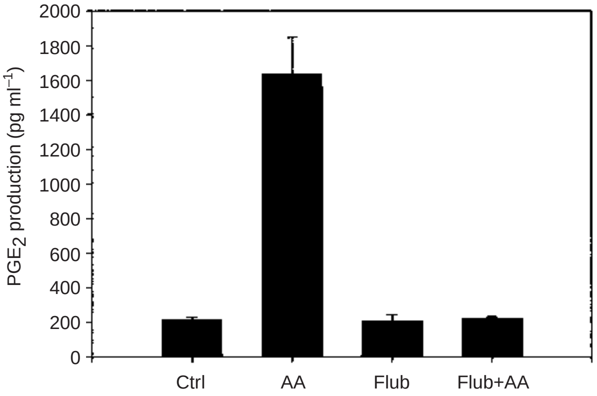

**Figure 2** NSAID inhibits PGE$_2$ production. Culture medium samples were collected prior to RNA isolation for the experiment described in Figure 1. Each bar represents the mean ± s.d. of three independent samples. Differences in PGE$_2$ levels were not significant except for the treatment with AA alone ($P < 0.001$)

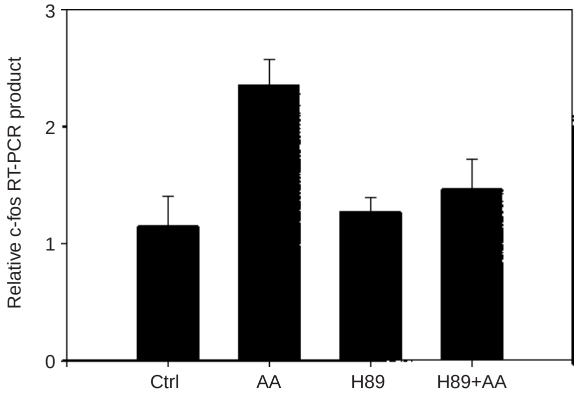

**Figure 3** PKA inhibitor inhibits AA-induced *c-fos* expression. The PKA specific inhibitor, H-89, was added to the cells at 30 μM 1 h before addition of AA. RNA was isolated from the cells 1 hour after addition of AA (1 μg ml$^{-1}$). Amount of *c-fos* RT-PCR product was normalized to the internal control, cyclophilin, and expressed as mean ± s.d. *c-fos* induction by AA was significant ($P < 0.001$) without H-89, but was not significant ($P = 0.713$) in presence of H-89

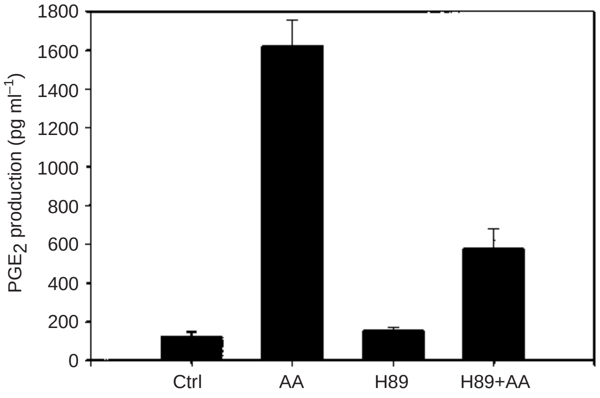

**Figure 4** Effect of H-89 on PGE$_2$ production from AA. Culture medium samples collected from the experiment described in Figure 3 were assayed for PGE$_2$ levels. Each bar represents mean ± s.d. of three independent samples. Increase of PGE$_2$ production by AA was significant in absence ($P < 0.001$) or presence ($P = 0.002$) of H-89

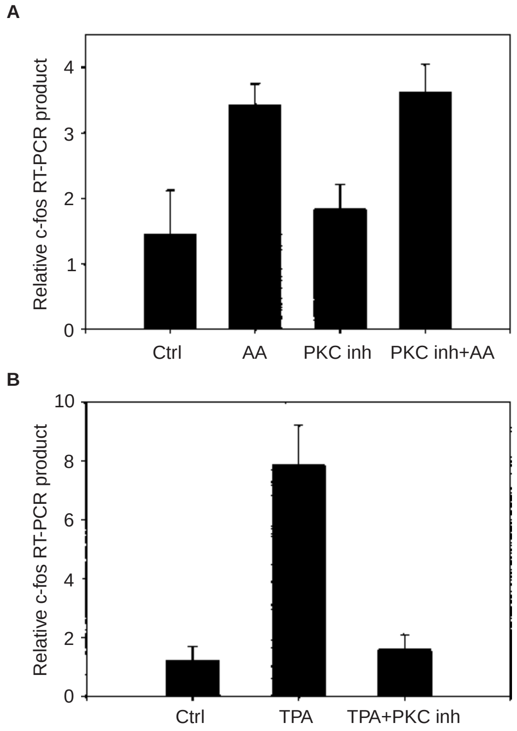

**Figure 5** PKC inhibitor has no effect on AA-induced *c-fos* expression. The PKC specific inhibitor, GF109203X, was added to the cells at 500 nM 30 min before addition of AA (1 μg ml$^{-1}$). TPA (1.6 μM) was added 30 min before cells were harvested for RNA isolation. AA was added 1 h before harvest. Amount of *c-fos* RT-PCR product was normalized to the cyclophilin product, and expressed as mean ± s.d. (**A**) Effect of GF109203X on AA-induced *c-fos* expression. Induction by AA was significant in presence of GF109203X ($P = 0.007$). (**B**) GF109203X inhibits TPA-induced *c-fos* expression

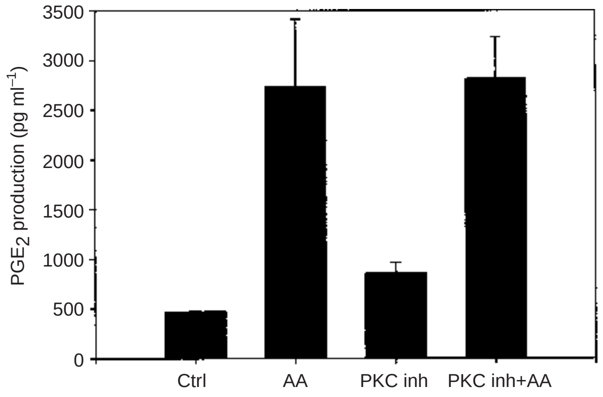

**Figure 6** PKC inhibitor does not affect PGE$_2$ production from AA. Culture medium samples collected from the experiment described in Figure 5A were assayed for PGE$_2$ levels. Each bar represents mean ± s.d. of three independent samples. Increase of PGE$_2$ production by AA was significant in absence ($P < 0.001$) or presence ($P = 0.002$) of GF109203X

involved in the AA-induced *c-fos* expression in the PC-3 cells, specific protein kinase inhibitors were used to treat the cells before AA was added. Treatment with H-89, a selective inhibitor of PKA (Chijiwa et al, 1990), resulted in loss of *c-fos* induction by AA (Figure 3). Inhibition of PKA also lowered PGE$_2$ production by 2.8-fold after AA was added to the cells (Figure 4). This reduced

*© 2000 Cancer Research Campaign*

$PGE_2$ level, however, was still 4.6-fold higher than the control samples, or 3.7-fold higher than the samples treated with H-89 but not with AA.

The specific PKC inhibitor, GF109203X (Toullec et al, 1991), was added to the cells prior to AA treatment in order to determine whether PKC is involved in *c-fos* induction in PC-3 cells. Figure 5A shows that inhibition of PKC did not significantly block AA-induced *c-fos* expression. The inhibitory effect of GF109203X was demonstrated by its strong inhibition of *c-fos* expression induced by TPA, which is a potent activator of PKC (Figure 5B). AA-induced $PGE_2$ production in PC-3 cells was also not affected by treatment with GF109203X (Figure 6).

Taken together, the results suggest that the PKA but not PKC signalling pathway is responsible for $PGE_2$ signal transduction that leads to increased *c-fos* expression after addition of AA. Therefore, the EP1 receptor, which activates the PKC pathway, is not likely to be involved in receiving the $PGE_2$ signal in PC-3 cells.

### Human prostate cells express the prostaglandin receptors EP4 and EP2

Expression of different EP receptors was examined in three human prostate cancer cell lines (PC-3, LNCaP, and DU145) and in PrEC normal prostate cells. Cells were grown in their normal growth media before harvested for RNA isolation and RT-PCR. A strong band was detected when the RT samples were PCR-amplified with EP4 primers after 31 cycles. This band was present in all four cell lines examined. Using the EP2 primers, only a weak band was present in all the cells after 40 cycles of amplification, suggesting a very low copy number. No RT-PCR product of the expected size was detected in any of the cell lines when EP1 or EP3 primers were used (Figure 7), even after varying the magnesium concentrations in PCR (data not shown). We conclude that the human prostate cells express EP4 and EP2 receptors and probably not EP1 or EP3.

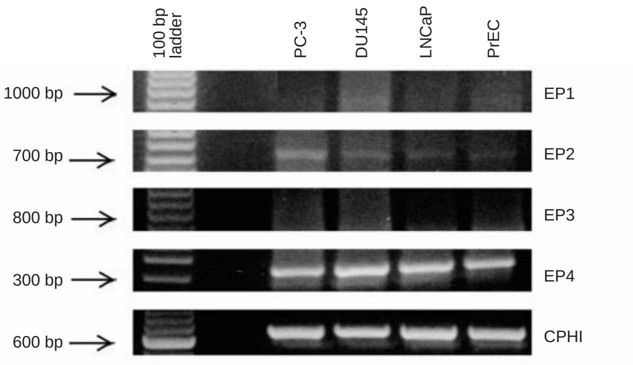

**Figure 7** EP receptor expression in human prostate cell lines. Total RNA from the PC-3, DU145, LNCaP and PrEC cells grown in their regular growth media were subjected to RT-PCR using specific EP primers. The PCR products were separated on agarose gels, and the portions of the gel corresponding to the expected DNA size of the PCR products are shown in the middle of the figure. The bands in the 100 bp ladder immediately below the expected PCR products are indicated by the arrows at left. The cyclophilin (CPHI) product is included in this figure as an internal control. PCR cycle numbers for the different primer pairs are: cyclophilin – 20 cycles; EP4 – 31 cycles; EP1, EP2, and EP3 – 40 cycles

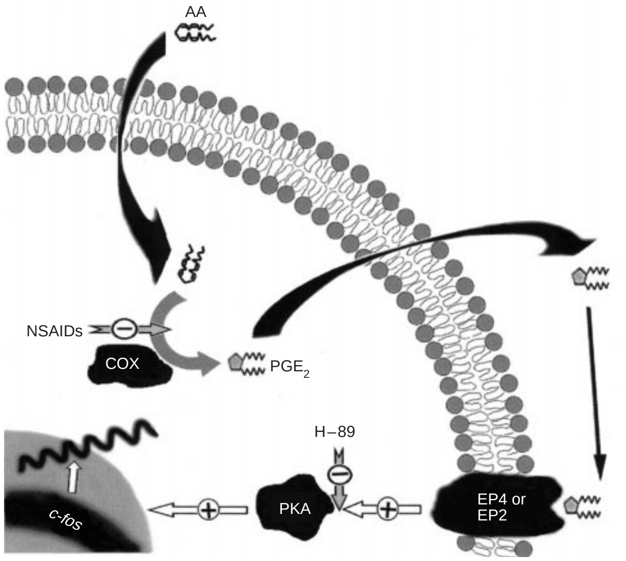

**Figure 8** Mechanism involved in increased *c-fos* expression by AA in PC-3 cells. AA is transported inside the cell and catalysed by the COX enzymes into $PGE_2$. $PGE_2$ is then secreted from the cells and binds to the EP4 and/or EP2 receptors. The EP4 or EP2 receptors activate the PKA pathway which sends signals inside the nucleus to induce *c-fos* gene transcription. Inhibition of the COX enzymes with NSAID (e.g. flurbiprofen) or inhibition of PKA with H-89 interrupts the signals from AA to *c-fos* induction

### DISCUSSION

The current study suggests that the mechanism by which AA increases *c-fos* expression and growth in PC-3 cells involves production of $PGE_2$ from AA, binding of $PGE_2$ to the EP4 and/or EP2 receptors, and subsequent activation of the PKA pathway which eventually leads to expression of growth-related genes (Figure 8). Under normal physiological conditions, AA is released from membrane phospholipids by phospholipases upon stimulation to synthesize eicosanoids (Smith, 1989). Because human cells can not synthesize AA de novo, AA has to be obtained directly from the diet, or synthesized from linoleic acid, which is also an essential fatty acid. AA is then present in serum either as an albumin-bound acid, or as part of lipids present in lipoproteins. Habenicht et al (1990) have demonstrated that cholesteryl-AA is taken up by the cells via the LDL receptor pathway. Serum albumin-bound AA enters the cells by endocytosis (Geuskens et al, 1994; Iturralde et al, 1991; Uriel et al, 1994). After transportation into the cells, AA is incorporated into the lipid pool as part of phospholipids (Chilton et al, 1996). The mechanisms by which prostaglandins (synthesized from AA via the cyclo-oxygenase pathway) facilitate cancer transformation include generation of mutagens, stimulation of cell growth, tumour promotion and immune suppression (Marnett, 1992). The importance of the cyclo-oxygenase products in prostate cancer has also been demonstrated by a recent study (Liu et al, 1998) in which a specific COX-2 inhibitor induced apoptosis of the LNCaP human prostate cell line.

*c-fos* is one of the earliest induced growth response genes (Lau and Nathans, 1987; Angel and Karin, 1991). In this study, expression of *c-fos* measured by semi-quantitative RT-PCR increased > 1.9-fold when prostate cancer cells were treated with AA in all experiments. The increased expression of *c-fos* may, at least in part, account for the increased PC-3 cell growth as previously observed (Tjandrawinata et al, 1997). Our results strongly suggest that AA induces *c-fos* expression through the biological activity of

 *© 2000 Cancer Research Campaign*

its product $PGE_2$ for two reasons. First, $PGE_2$ had a similar effect on *c-fos* expression in PC-3 cells; and second, treatment with a cyclo-oxygenase inhibitor suppressed the induction of *c-fos* expression by AA. To understand the signal transduction pathway(s) involved in AA-induced *c-fos* expression in the prostate cancer cells, specific protein kinase inhibitors were used. The results clearly indicate a role of PKA, but not PKC, in the activation of the signal transduction event. Therefore, the prostaglandin receptor(s) that mediate this event must be EP2 and/or EP4, which are linked to the PKA pathway. Consistent with the results from protein kinase inhibition experiments, RT-PCR detected EP4 as the major EP receptor expressed in human prostate cells, with a small amount of EP2 expression also detected. Expression of EP1 and EP3 was not detected. Expression of specific types of EP receptors appears to be the mechanism used by different cell types for carrying out various physiological functions of the eicosanoids.

Interestingly, the PKA inhibitor H-89 partially inhibited $PGE_2$ production from AA. This, however, was unlikely the cause for the complete loss of AA-induction of *c-fos* expression, because the $PGE_2$ level in presence of H-89 and AA was still much higher than in control samples. Two possibilities are proposed to explain this partial inhibition of $PGE_2$ synthesis. First, COX-2 is a feed-forward enzyme up-regulated by its product $PGE_2$ (Pilbeam et al, 1995; Tjandrawinata et al, 1997). Inhibition of PKA blocks the pathway by which $PGE_2$ exerts its cellular functions through the EP4/2 receptors in PC-3 cells. This presumably prevents further activation of cyclo-oxygenases, thus decreasing the amount of subsequent $PGE_2$ production. Second, it is possible that other stimulating factors are needed for conversion of AA into $PGE_2$, and the PKA inhibitor partially blocked the transmission of such signals. In our study, we also demonstrated *c-fos* inducibility by the phorbol ester TPA, which is a potent PKC activator and mitogen. The fact that activation of PKC in absence of the EP1 receptor induced *c-fos* further supported the role of EP receptors as the decisive control for the different responses to eicosanoids.

The *c-fos* promoter contains a serum response element (SRE), which is activated by signals through the PKC pathway (Buscher et al, 1988; Gilman, 1988). A cyclic AMP response element (CRE) is also located at −64/−57 of the *c-fos* promoter. In mouse osteoblasts which also depend on the PKA pathway for $PGE_2$-induced *c-fos* expression, the CRE but not the SRE has been suggested to provide the inducibility by $PGE_2$ (Fitzgerald et al, 1999). In that study the *c-fos* promoter with 5′ deletions was fused to a reporter gene and the effect of exogenous $PGE_2$ on promoter activity studied. With the findings presented here, the CRE is also expected to confer $PGE_2$-inducibility in the PC-3 cells.

This report has elucidated several key steps by which certain dietary fatty acids, such as AA and its precursors, increase human prostate cell growth and cancer risk. Our results strongly suggest that $PGE_2$, the EP4/2 receptors, and the PKA pathway are mediators of AA induced *c-fos* expression in the human prostate cancer cell line PC-3.

### ACKNOWLEDGEMENTS

This work was supported by the Department of Veteran's Affairs, VAMC, San Francisco, and by NASA grant NAG-2-1086. The authors thank Dr Rajvir Dahiya for providing the LNCaP cell line. We thank Vicki Gilbertson for the comments on the manuscript and technical guidance in RT-PCR. Angela Wong provided excellent technical assistance.

### REFERENCES

Angel P and Karin M (1991) The role of Jun, Fos and the AP-1 complex in cell-proliferation and transformation. *Biochim Biophys Acta* **1072**: 129–157

Buscher M, Rahmsdorf HJ, Litfin M, Karin M and Herrlich P (1988) Activation of the c-fos gene by UV and phorbol ester: different signal transduction pathways converge to the same enhancer element. *Oncogene* **3**: 301–311

Chaudry AA, Wahle KW, McClinton S and Moffat LE (1994) Arachidonic acid metabolism in benign and malignant prostatic tissue in vitro: effects of fatty acids and cyclooxygenase inhibitors. *Int J Cancer* **57**: 176–180

Chijiwa T, Mishima A, Hagiwara M, Sano M, Hayashi K, Inoue T, Naito K, Toshioka T and Hidaka H (1990) Inhibition of forskolin-induced neurite outgrowth and protein phosphorylation by a newly synthesized selective inhibitor of cyclic AMP-dependent protein kinase, N-[2-(p-bromocinnamylamino)ethy1]-5-isoquinolinesulfonamide (H-89), of PC12D pheochromocytoma cells. *J Biol Chem* **265**: 5267–5272

Chilton FH, Fonteh AN, Surette ME, Triggiani M and Winkler JD (1996) Control of arachidonate levels within inflammatory cells. *Biochim Biophys Acta* **1299**: 1–15

Coleman RA and Kennedy I (1985) Characterisation of the prostanoid receptors mediating contraction of guinea-pig isolated trachea. *Prostaglandins* **29**: 363–375

Connolly JM, Coleman M and Rose DP (1997) Effects of dietary fatty acids on DU145 human prostate cancer cell growth in athymic nude mice. *Nutr Cancer* **29**: 114–119

Danesch U, Weber PC and Sellmayer A (1994) Arachidonic acid increases c-fos and Egr-1 mRNA in 3T3 fibroblasts by formation of prostaglandin E2 and activation of protein kinase C. *J Biol Chem* **269**: 27258–27263

Fitzgerald J, Dietz TJ and Hughes-Fulford M (2000) Prostaglandin $E_2$-induced upregulation of *c-fos* mRNA is primarily mediated by cAMP in MC3T3-E1 osteoblasts. *Endocrinology* **141**: 291–298

Geuskens M, Torres JM, Esteban C and Uriel J (1994) Endocytosis of three serum proteins of a multigene family and of arachidonic acid in human lectin-stimulated T lymphocytes. *Microsc Res Tech* **28**: 297–307

Gilman MZ (1988) The c-fos serum response element responds to protein kinase C-dependent and -independent signals but not to cyclic AMP. *Genes Dev* **2**: 394–402

Habenicht AJ, Salbach P, Goerig M, Zeh W, Janssen-Timmen U, Blattner C, King WC and Glomset JA (1990) The LDL receptor pathway delivers arachidonic acid for eicosanoid formation in cells stimulated by platelet-derived growth factor. *Nature* **345**: 634–636

Iturralde M, Alava MA, Gonzalez B, Anel A and Pineiro A (1991) Effect of alpha-fetoprotein and albumin on the uptake of polyunsaturated fatty acids by rat hepatoma cells and fetal rat hepatocytes. *Biochim Biophys Acta* **1086**: 81–88

Juzan M, Hostein I and Gualde N (1992) Role of thymus-eicosanoids in the immune response. *Prostaglandins Leukot Essent Fatty Acids* **46**: 247–255

Kacich RL, Williams LT and Coughlin SR (1988) Arachidonic acid and cyclic adenosine monophosphate stimulation of c-fos expression by a pathway independent of phorbol ester-sensitive protein kinase C. *Mol Endocrinol* **2**: 73–77

Lau LF and Nathans D (1987) Expression of a set of growth-related immediate early genes in BALB/c 3T3 cells: coordinate regulation with c-fos or c-myc. *Proc Natl Acad Sci USA* **84**: 1182–1186

Liu XH, Yao S, Kirschenbaum A and Levine AC (1998) NS398, a selective cyclooxygenase-2 inhibitor, induces apoptosis and down-regulates bcl-2 expression in LNCaP cells. *Cancer Res* **58**: 4245–4249

Marnett LJ (1992) Aspirin and the potential role of prostaglandins in colon cancer. *Cancer Res* **52**: 5575–5589

Mehmet H, Millar JB, Lehmann W, Higgins T and Rozengurt E (1990) Bombesin stimulation of c-fos expression and mitogenesis in Swiss 3T3 cells: the role of prostaglandin E2-mediated cyclic AMP accumulation. *Exp Cell Res* **190**: 265–270

Monick M, Glazier J and Hunninghake GW (1987) Human alveolar macrophages suppress interleukin-1 (IL-1) activity via the secretion of prostaglandin E2. *Am Rev Respir Dis* **135**: 72–77

Negishi M, Sugimoto Y and Ichikawa A (1995) Molecular mechanisms of diverse actions of prostanoid receptors. *Biochim Biophys Acta* **1259**: 109–119

O'Neill GP and Ford-Hutchinson AW (1993) Expression of mRNA for cyclooxygenase-1 and cyclooxygenase-2 in human tissues. *FEBS Lett* **330**: 156–160

Pilbeam CC, Raisz LG, Voznesensky O, Alander CB, Delman BN and Kawaguchi H (1995) Autoregulation of inducible prostaglandin G/H synthase in osteoblastic cells by prostaglandins. *J Bone Mineral Res* **10**: 406–414

Raisz LG, Pilbeam CC and Fall PM (1993) Prostaglandins: mechanisms of action and regulation of production in bone. *Osteoporos Int* **3 (Suppl 1)**: 136–140

Rao GN, Lassegue B, Griendling KK, Alexander RW and Berk BC (1993) Hydrogen peroxide-induced c-fos expression is mediated by arachidonic acid release: role of protein kinase C. *Nucleic Acids Res* **21**: 1259–1263

Rose DP (1997) Dietary fatty acids and cancer. *Am J Clin Nutr* **66(Suppl)**: 998S–1003S

Rose DP and Connolly JM (1991) Effects of fatty acids and eicosanoid synthesis inhibitors on the growth of two human prostate cancer cell lines. *Prostate* **18**: 243–254

Smith WL (1989) The eicosanoids and their biochemical mechanisms of action. *Biochem J* **259**: 315–324

Tjandrawinata RR, Dahiya R and Hughes-Fulford M (1997) Induction of cyclo-oxygenase-2 mRNA by prostaglandin E2 in human prostatic carcinoma cells. *Br J Cancer* **75**: 1111–1118

Toullec D, Pianetti P, Coste H, Bellevergue P, Grand-Perret T, Ajakane M, Baudet V, Boissin P, Boursier E, Loriolle F, Duhamel L, Charon D and Kirilovsky J (1991) The bisindolylmaleimide GF 109203X is a potent and selective inhibitor of protein kinase C. *J Biol Chem* **266**: 15771–15781

Uriel J, Torres JM and Anel A (1994) Carrier-protein-mediated enhancement of fatty-acid binding and internalization in human T-lymphocytes. *Biochim Biophys Acta* **1220**: 231–240

Weinreb M, Grosskopf A and Shir N (1999) The anabolic effect of PGE2 in rat bone marrow cultures is mediated via the EP4 receptor subtype. *Am J Physiol* **276**: E376–383

 *© 2000 Cancer Research Campaign*
